# Congrong Shujing Granule-Induced GRP78 Expression Reduced Endoplasmic Reticulum Stress and Neuronal Apoptosis in the Midbrain in a Parkinson's Disease Rat Model

**DOI:** 10.1155/2020/4796236

**Published:** 2020-09-30

**Authors:** Qian Xu, Shasha Yang, Fangzhen Wu, Yao Lin, Jianan Zhong, Lanfang Tang, Xuefeng Hu, Jing Cai

**Affiliations:** ^1^Fujian Key Laboratory of Developmental and Neural Biology & Southern Center for Biomedical Research, College of Life Science, Fujian Normal University, Fuzhou, Fujian 350117, China; ^2^College of Integrative Medicine, Fujian University of Traditional Chinese Medicine, Fuzhou, Fujian 350122, China; ^3^Department of Rheumatology and Endocrinology, The Second Affiliated Hospital of Fujian Traditional Chinese Medical University, Fuzhou 350003, China

## Abstract

The main pathological changes inherent in Parkinson's disease (PD) are degeneration and loss of dopamine neurons in the midbrain and formation of Lewy bodies. Many studies have shown that the pathogenesis of PD is closely related to endoplasmic reticulum (ER) oxidative stress. This study combined various traditional Chinese medicines to prepare Congrong Shujing granules (CSGs). The optimal dose combination of the ingredients was identified by experimental intervention in SH-SY5Y cells *in vitro*. A PD rat model was established by intraperitoneal injection of rotenone sunflower oil emulsion. The suspension tests were performed on the 14th day after modeling and also on the 14th day after CSG intervention (5.88 g/kg, 11.76 g/kg, and 23.52 g/kg). We evaluated the changes in motor function and the expression of neuronal cell functional marker proteins, ER stress (ERS) marker proteins, and apoptosis-related pathway proteins of neuronal cells. Changes in cellular ultrastructure were observed by electron microscopy. Our results showed that CSG treatment lengthened the duration of PD rats' gripping to the wire. 78 kDa glucose-regulated protein (GRP78) expression in the substantia nigra was significantly upregulated in the middle- and high-dose CSG groups after 14 days of treatment compared with the model group. The expression of the key dopaminergic neuron functional enzyme tyrosine hydroxylase (TH) and cerebral dopamine neurotrophic factor (CDNF) was elevated. The expression of c-Jun N-terminal kinase (JNK) and phosphorylated c-Jun decreased, and cell apoptosis was significantly reduced. Compared with the model group, the treatment groups had fewer ER fragmentation and degranulation (ribosome shedding) and abundant ER and mitochondria suggesting that CSG reduced ER stress and neuronal apoptosis in the midbrain of a PD rat model by inducing the expression of molecular chaperone GRP78.

## 1. Introduction

Neurodegenerative disease has become the third most fatal disease globally after cardiovascular and cerebrovascular diseases and cancer, and Parkinson's disease (PD) is the third most common neurodegenerative disease. In China, the prevalence of PD among those over 65 years of age is approximately 1.7% and is expected to affect approximately 5 million patients by 2030. Symptoms such as tremor, bradykinesia, rigidity, and ataxia gradually aggravate with the disease course, which lead to the loss of independent living and a financial burden for the family and society.

The main pathological features of PD are the progressive loss of dopamine neurons, apoptosis, and abnormal aggregation of proteins (Lewy bodies). *α*-Syn is the main component of Lewy bodies. Many studies have shown that the abnormal aggregation of *α*-syn promotes the release of cerebral inflammatory response and neurotoxic substances, thereby disrupting the homeostasis of endoplasmic reticulum (ER) to cause ER stress (ERS) and induce neuronal apoptosis [1–3].

ERS refers to the disruption of ER morphology and functional homeostasis in the cells by internal and external factors resulting in an accumulation of unfolded or misfolded proteins in the ER. In response, cells initiate an unfolded protein response (UPR) to change the cellular transcription and translation levels. The burden of the cells is relieved by lowering the level of protein synthesis and increasing the expression of various molecular chaperones to assist protein folding [4, 5]. Programmed cell death is initiated if the ERS is strong enough. If appropriate intervention is performed at the same time, the ERS will be reduced and the cells will resume to the homeostatic state [6].

Sustained and excessively strong ERS induces apoptosis by activating the three major downstream apoptotic signaling pathways [7]: (1) inositol-requiring enzyme-1-tumor necrosis factor receptor-associated factor-2-apoptosis signal-regulating kinase-1-c-Jun *N*-terminal kinase (IRE-1-TRAF2-ASK1-JNK)-related pathway, (2) caspase-12-related pathway, and (3) protein kinase RNA-like ER kinase-eukaryotic initiation factor-2 alpha-activating transcription factor-4-CCAAT enhancer binding protein homologous protein (PERK-eIF2a-ATF4-CHOP)-related pathway. Under ERS conditions, JNK is activated after phosphorylation further activating the c-JUN activity to induce apoptosis; alternatively, the calcium ion concentration is increased to induce caspase-12-mediated apoptosis. The UPR counters ERS by increasing the expression of molecular chaperone 78 kDa glucose-regulated protein (GRP78). Marker molecules such as IRE-1 and PERK, which are involved in the UPR, usually prompt the occurrence of ERS. Both IRE-1 and PERK bind to the molecular chaperone GRP78. If GRP78 is highly expressed, it does not dissociate from ER transmembrane proteins PERK and IRE-1, which cannot undergo dimerization and autophosphorylation, failing to trigger the JNK apoptosis pathway. Therefore, high GRP78 expression reduces ERS and the expression of apoptosis-related proteins, rescuing cell survival. Neurotrophic factor family proteins, such as mesencephalic astrocyte-derived neurotrophic factor (MANF) and cerebral dopamine neurotrophic factor (CDNF), which also bind to GRP78, have 59% amino acid homology [8]. Studies have shown that CDNF protects the growth of neuron axons and promotes neuroregeneration and functional recovery of nerves [9–11].

Traditional Chinese medicine (TCM) has been widely used as an adjuvant therapy to PD treatment due to its relatively mild side effects and possible broader pharmacological effects. Congrong Shujing granule (CSG) is based on a combination of a TCM classic formula and clinical experience and has obtained a national patent (Patent number: 201410658358.3) in China. It originates from a classic TCM prescription, Dihuang Yinzi, recorded in the ancient Chinese medical book *Xuanming Lun Fang* (*Clear Synopsis on Recipes*), also named *Yellow Emperor's Questions of Clear Synopsis on Recipes*, written by Liu Wansu in 1172 AD. CSG consists of five herbs: *Cistanche deserticola*, refined *Rhizoma polygonati*, *Salvia miltiorrhiza*, *Radix Paeoniae Rubra*, and tree peony bark. *C. deserticola* is considered to be the principal component in the prescription in TCM theory, boosting kidney Yang and nourishing essence and blood. Modern pharmacology has shown that *C. deserticola* increases the content of neurotransmitters and improves the motor function in a PD mouse model [12]. Its active ingredient, echinacoside, significantly inhibits neuronal apoptosis, increases neural stem cell proliferation in the dentate gyrus of PD rats, and reduces the loss of dopaminergic neurons induced by 6-hydroxydopamine (6-OHDA) [13]. The *S. miltiorrhiza*, *R. Paeoniae Rubra*, and tree peony bark promote blood circulation, eliminating blood stasis and improving vascular function and microcirculation [14]. However, the pharmacological mechanisms of CSG compound have not been fully elucidated.

This study aims to evaluate the effect of CSG on ERS and neuronal apoptosis in PD *in vitro* and *in vivo*. First, the effective dose combination was evaluated in a nerve cell culture. Subsequently, the phenotypic changes after treatment were evaluated in a PD rat model. The expression of neuronal cell functional marker proteins, ERS marker proteins, and apoptosis-related pathway proteins was measured to evaluate the ERS levels and neuronal apoptosis improved by CSG.

## 2. Methods

### 2.1. In Vitro Screening of CSG Dose Combinations

In this study, two dose levels of each of the five herbs including *C. deserticola*, refined *R. polygonati*, *S. miltiorrhiza*, *R. Paeoniae Rubra*, and tree peony bark were tested. An orthogonal design was used to evaluate the effects on neuronal apoptosis *in vitro* to identify the optimal dose combination. All medicinal materials were purchased from Beijing Tong Ren Tang Chinese Medicine Company Limited (Beijing, China) and identified by Professor Dan Chen of the School of Pharmacy, Fujian University of TCM, Fujian Province, China. The L8 (2^7^) orthogonal table (Table 1) was used to place the above five herbs in each column, and experimental intervention was performed in the cells with different dose combinations of the herbs. The herbs in high and low doses were combined according to the orthogonal table to form prescriptions of different dose combinations. Distilled water was added to submerge the herbs before boiling and preparing the decoction, which was filtered to remove herbal residue. The filtered decoction was subsequently concentrated by rotary evaporator to a final concentration of 100 *μ*g/ml.

### 2.2. Cell Culture and Viability Assay

Human neuroblastoma SH-SY5Y cells were purchased from the Shanghai Cell Bank of Chinese Academy of Sciences (Shanghai, China). Complete medium contained 445 ml DEME/F12, 50 ml fetal bovine serum, and 5 ml penicillin and streptomycin). Once the cells grew to 80–90% confluence, they were washed once with phosphate buffered saline (PBS) and incubated with 1 ml trypsin in the incubator after discarding the PBS. The complete medium was added to stop the reaction 2 min after the trypsinization, followed by pipetting the cells into a suspension and centrifuging at 1,500 rpm for 5 min. After centrifugation and supernatant removal, the cell pellet was resuspended in the complete medium and aliquoted into new culture flasks. The cells were divided into control, PD model, and drug intervention groups (compounds A–H). Cell viability was detected using the MTT assay after trypsinization and preparing a single-cell suspension in each group. By inoculating a density of 3,000 cells per well in a 96-well plate and preparing five replicates per group, MPP^+^ solution (1 mmol/ml) was added to each group after 24 h, followed by the addition of aqueous solution of different dose combinations to the cells for 24 h. After another 24 h, 20 *μ*l 5 mg/ml MTT in PBS (pH 7.4) was added to each well, followed by incubation of the cells at 37°C for 4 h. After the supernatant was removed, 150 *μ*l DMSO was added to each well to react on a shaker for 10 min until the crystals were sufficiently dissolved. The assay was subsequently analyzed by a microplate reader to read the absorbance (OD value) at a wavelength of 570 nm: cell viability = (OD of the drug intervention group/OD of the model group) × 100%.

### 2.3. Animal Modeling and Drug Treatment

A total of 70 specific-pathogen-free (SPF) male Sprague-Dawley (SD) rats, weighing 180 ± 20 g, were kept at the SPF-grade Experimental Animal Center of Fujian University of TCM, Fujian Province, China.

The rats were randomly divided into control, solvent, and PD model groups. Rats in the PD model group were intraperitoneal injected with rotenone and sunflower oil emulsion (1.5 mg/kg/day) for 14 days to establish a PD model, which was ranked according to the scoring criteria of the PD model. Rats with a score of ≥2 were selected as PD rats. Rats in the solvent group were subcutaneously injected with an equal volume of sunflower oil emulsion for 14 days. Then, the PD rats were randomly divided into a model group, low-dose group, middle-dose group, and high-dose group. The low-dose, the middle-dose, and the high-dose groups were treated by instillation with CSG once a day for 14 days. Meanwhile, the rats in the control, solvent, and model groups were given the same volume of normal saline for 14 days.

Experimental CSG daily dose in rat was calculated according to the daily dosage of 112 g in human and the human-rat body surface area ratio [15] (human daily dose/60 kg × 6.3 = middle dose in rats). The calculated daily dosages for rats in the low-, middle-, and high-dose groups were 5.88 g/kg, 11.76 g/kg, and 23.52 g/kg, respectively. The herbal materials were mixed according to the dose combination and submerged in distilled water (level just covered the herbs) to boil and prepare the decoction, which was filtered to remove herbal residue. The filtered decoction was subsequently concentrated by rotary evaporator to a final volume of 1 ml. The concentrated decoctions of low-, middle-, and high-dose groups contained 0.0588 g, 1.176 g, and 2.352 g CSG, respectively, which were intragastrically administered according to the bodyweight of 100 g/ml in the corresponding rats of the low-, middle-, and high-dose drug intervention groups.

### 2.4. Behavioral Analyses

On the 14th day after modeling (before intervention) and the 14th day after CSG intervention, the suspension test was performed in the rats of each group. Rats were suspended on a horizontally straightened steel wire with a diameter of approximately 3 mm and a distance of 30 cm from the ground. A soft pad was placed on the ground to prevent injury from falling. During the test, the rats were not allowed to sit on the wire, and both forelimbs of the rats had to be suspended from the wire. In addition, the condition of front paws gripping on the wire and the duration of wire hanging of each rat was recorded and scored, with wire gripping continuously for 0–5 s scored as 0, 6–10 s as 1, 11–15 s as 2, 16–20 s as 3, 21–25 s as 4, 26–30 s as 5, and >30 s as 6. Scoring of each animal was recorded three times to take the mean value with an interval of approximately 2 min between each test.

### 2.5. Detection of Relevant Protein Expression

The rats were anesthetized with urethane (1000 mg/kg bodyweight) by intraperitoneal injection, and no pain or discomfort was observed. This was because the anesthetic caused muscle relaxation and its other effects were mild. Rats were sacrificed by rapid cervical dislocation. Animals had little anxiety or pain by this way. We gave full consideration to the interests of animals; treated them humanely; prevented or reduced their stress, pain, and injury; respected animal life; stopped the barbarism against animals; and adopted the least painful method to deal with animals. The methods and purposes of animal experiments were in conformity with human ethical standards and international practices. Brain tissues of three rats from each group were collected for the detection of protein expression by rinsing off the fixative on the tissues and cryosectioning into brain slices.

In addition, three rats of each group were anesthetized intraperitoneally and perfused with 4% paraformaldehyde solution. After the perfusion was completed, the whole brain of each animal was carefully isolated and embedded in paraffin before sectioning. After washing, the precooled brain sections were washed three times in PBS, and the brain sections were blocked with goat serum for two hours. After discarding, the serum was discarded; anti-TH (1 : 400), anti-GRP78 (1 : 200), and anti-CDNF (1 : 200) primary antibodies were independently incubated with the brain sections at 4°C overnight. The remaining steps of the immunohistochemistry were performed in accordance with the manufacturer's instructions of the Maxim immunohistochemistry kit (Maxim Biotechnologies, Fujian Province, China). The brain sections were then developed in 3,3′-diaminobenzidine (DAB) color development solution, counterstained nuclei were counterstained in hematoxylin dye, and the excessive dye was removed in 1% hydrochloric acid alcohol. After dehydrating in gradient ethanol solutions (3 min each) and clearing in xylene solutions I and II (3 min each), the brain sections were mounted with neutral gum. Once the mounted brain sections were dried up, they were subjected to imaging under a microscope. The relative integral optical density (IOD) values of TH, GRP78, and CDNF and the total area of the sections was measured: the average optical density value of the target protein = the IOD value of the target protein/total area of the tested brain region in the section.

For Western blot analysis, the substantia nigra in the midbrain of each group of rats was isolated and weighed, followed by complete homogenization and lysis for target protein detection. The anti-*β*-actin mAb (Proteintech Group, 66009-1-lg), anti-GRP78/Bip mAb, (Abcam, ab21685), anti-CDNF mAb (Novus, NBP1-76834), anti-JNK mAb, (Abcam, ab126591), anti-c-JUN Rabbit mAb (Cell Signaling Technology, 9165), anti-Phospho-c-JUN mAb (Cell Signaling Technology, 91952), anti-IRE1*α* mAb (Abcam, ab37073), anti-Phospho-IRE1*α* mAb (Abcam, ab124945), and anti-Caspase-12 mAb (Abcam, ab62463) were used for Western blot analysis. The corresponding protein bands detected by Western blot analysis were analyzed semiquantitatively by the Image Lab software.

Then, the brain tissue of rats was taken out, and the frozen sections were obtained. The frozen sections were put in citric acid buffer (pH 6.0) for antigen repair and then incubated with primary antibody (anti-TH mAb (Abcam, ab137869); anti-GRP78/Bip mAb (Abcam, ab21685)) and secondary antibody (goat anti-Rabbit IgG secondary antibody, Proteintech Group, 10285-1-AP), with PBS rinsing and DAPI redyeing. The slices were dried by antifluorescence quenching agent and put in Nikon fluorescence microscope for image acquisition.

### 2.6. Observation of Neuron Numbers and Microstructure by Electron Microscopy

We observed the number of neurons in the midbrain using DAPI staining, and we used the results to quantify apoptosis of neurons. The brain slices were stained in DAPI solution by covering the section and incubating at room temperature for 10 min. After removing the DAPI solution, the brain slices were washed in PBS three times (3 min each), followed by fluorescent microscopy at an excitation wavelength of 360 nm. We counted the number of neurons in the same area of view and compared the results from different groups.

The tissue samples were fixed with 2.5% glutaraldehyde, followed by rinsing three times in 1 M PBS and fixing with 1% osmic acid for 1 h. After rinsing in PBS and dehydrating with acetone, the tissue samples were embedded and sectioned into ultrathin slices, which were first stained with lead citrate for 10 min and washed with carbon dioxide-free double-distilled water three times and uranium acetate for 30 min. After washing in double-distilled water for three times and drying, the stained ultrathin slices were imaged by transmission electron microscope (TEM).

### 2.7. Statistical Analyses

The experimental data were processed and analyzed by SPSS 24.0 (IBM SPSS Inc., Chicago, IL). The measurement data were presented as mean ± standard deviation. Comparison between multiple groups was performed by one-way analysis of variance (ANOVA). Analysis of the behavioral data was performed using a repeated measure ANOVA. Pairwise comparisons between groups were performed using Fisher's least significant difference (LSD) method or Games–Howell method. The test level was *α* = 0.05.

## 3. Results

**Figure 1:**
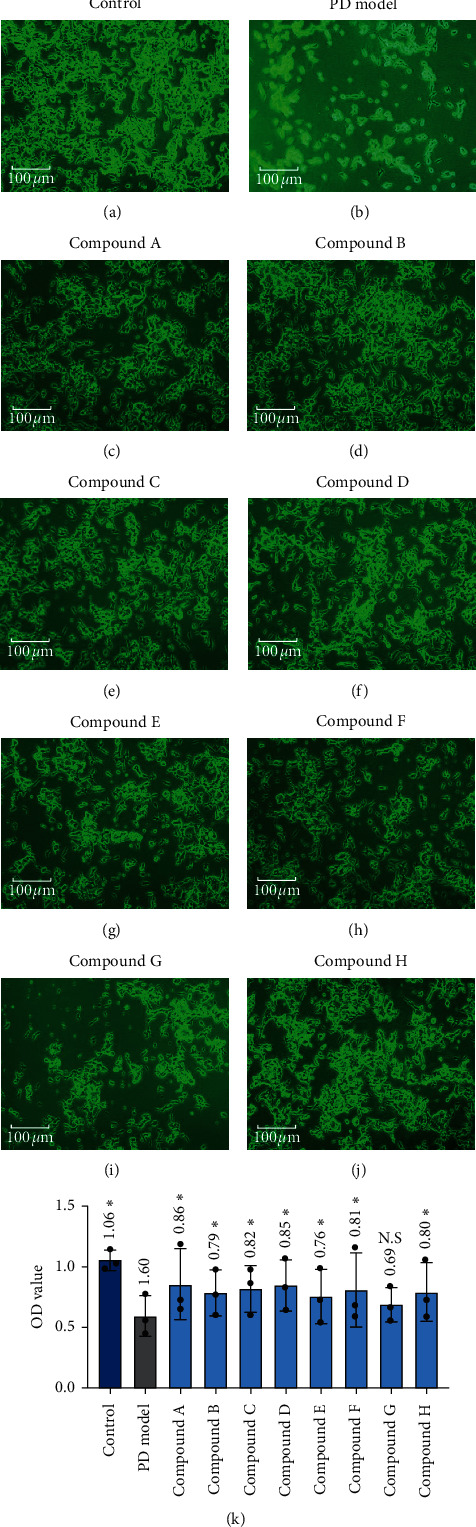
Morphology and OD value of the cells in the drug efficacy screening experiment. (a–j) The effects of different TCM compounds on SH-SY5Y cell survival. (k) The statistical data of OD values of the groups. Compared with the PD model, “^∗^” indicates *P* < 0.05 and “N.S” indicates no significant differences.

Table 1 shows the results of the drug orthogonal design, grouping, and results of average cell viability in this study. The effects of different dose combinations on MPP^+^-induced *in vitro* cell models are shown in Table 2 and Figure 1. The orthogonal design analysis revealed that the two tested doses of *C. deserticola*, *S. miltiorrhiza*, and tree peony bark had different therapeutic effects on the *in vitro* model (*P* < 0.05). They reduced apoptosis, maintained cell number, and promoted proliferation in vitro. *C. deserticola* dose 1, *S. miltiorrhiza* dose 2, and tree peony bark dose 1 had better effects compared to the other corresponding dose of the three herbs. By contrast, the different doses of refined *R. polygonati* and *R. Paeoniae Rubra* in the CSG exhibited similar therapeutic effects. Based on the above results and the principle of simplification, we selected the optimal experimental formula of 6 g *C. deserticola*, 12 g refined *R. polygonati*, 15 g *S. miltiorrhiza*, 12 g *R. Paeoniae Rubra*, and 10 g tree peony bark to prepare the CSG compound formula.

**Figure 2:**
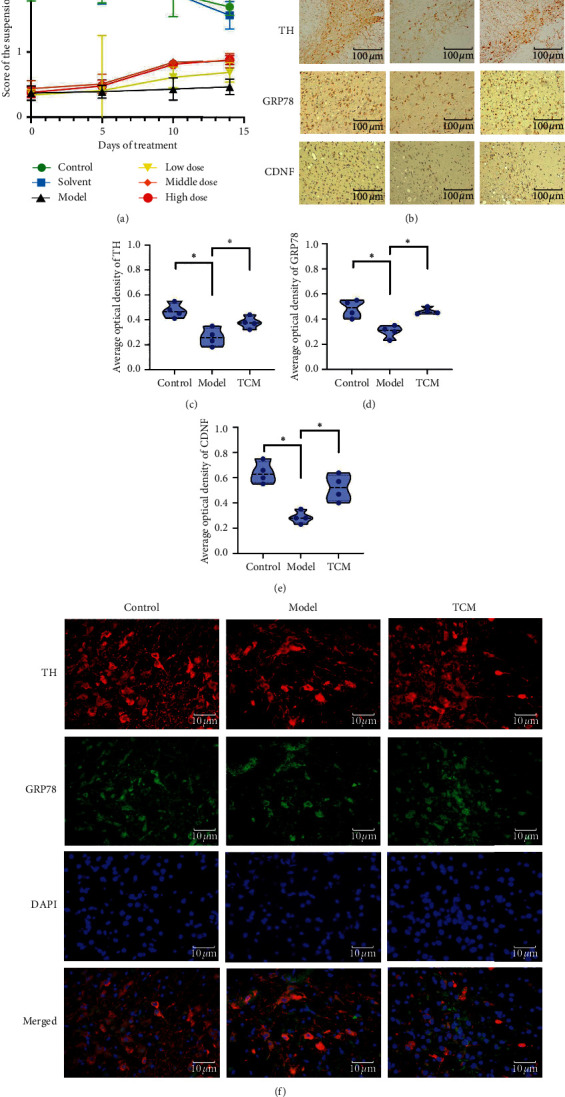
Ethology and immunostaining of rats. (a) Score of the suspension experiment after different days of treatment. (b–f) Expression of TH, GRP78, and CDNF in the substantia nigra. The “TCM group” refers to the middle-dose group. Compared with the model group, “^∗^” indicates *P* < 0.05.

As shown in Tables 3 and 4 and Figure 2(a) about the results of rat behavioral analyses in different groups, CSG had a certain effect in prolonging the duration of rat's gripping to the wire. The scores of the suspension experiment on different days (except the PD model group) and in different groups showed significant difference (*P* < 0.01).

**Figure 3:**
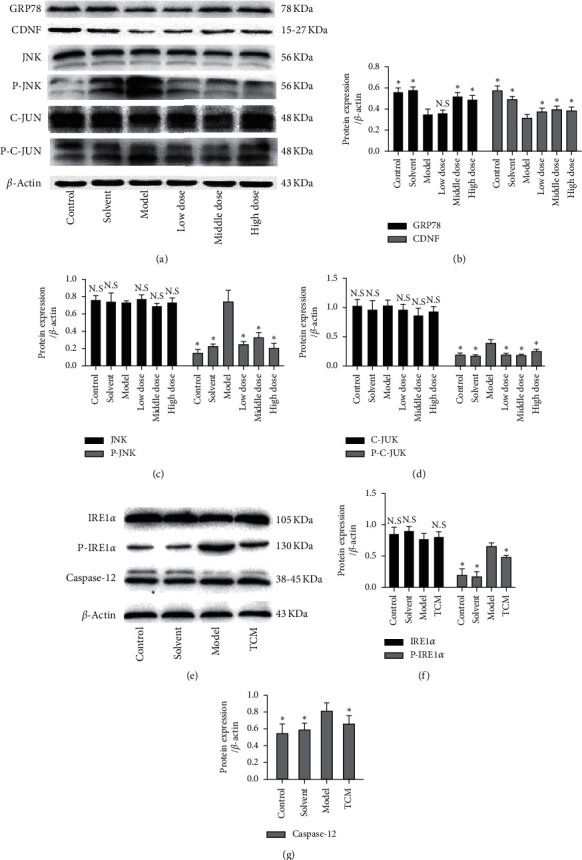
Protein expression in different groups as indicated by Western blot analysis. (a–d) Levels of expression of GRP78, CDNF, and JNK pathway-related proteins. (e–g) The levels of expression of IRE1*α* and caspase-12. Compared with the model group, “^∗^” indicates *P* < 0.05 and “N.S” indicates no significant differences.

Immunohistochemistry showed that CSG interventions increased the TH, GRP78, and CDNF expressions (Figures 2(b)–2(e)), and immunofluorescence labeling analysis demonstrates that GRP78 was expressed in TH-positive cells in substantia nigra (Figure 2(f)). And CSG decreased the JNK and c-JUN expression in rat substantia nigra, thereby reducing ERS and rescuing cell survival. To further test the hypothesis that CSG could reduce ERS and neuronal apoptosis, IRE1*α* and caspase-12 were also examined as markers of ERS and apoptosis. The results showed that CSG increased IRE1*α* phosphorylation and decreased caspase-12 expression (Figure 3).

**Figure 4:**
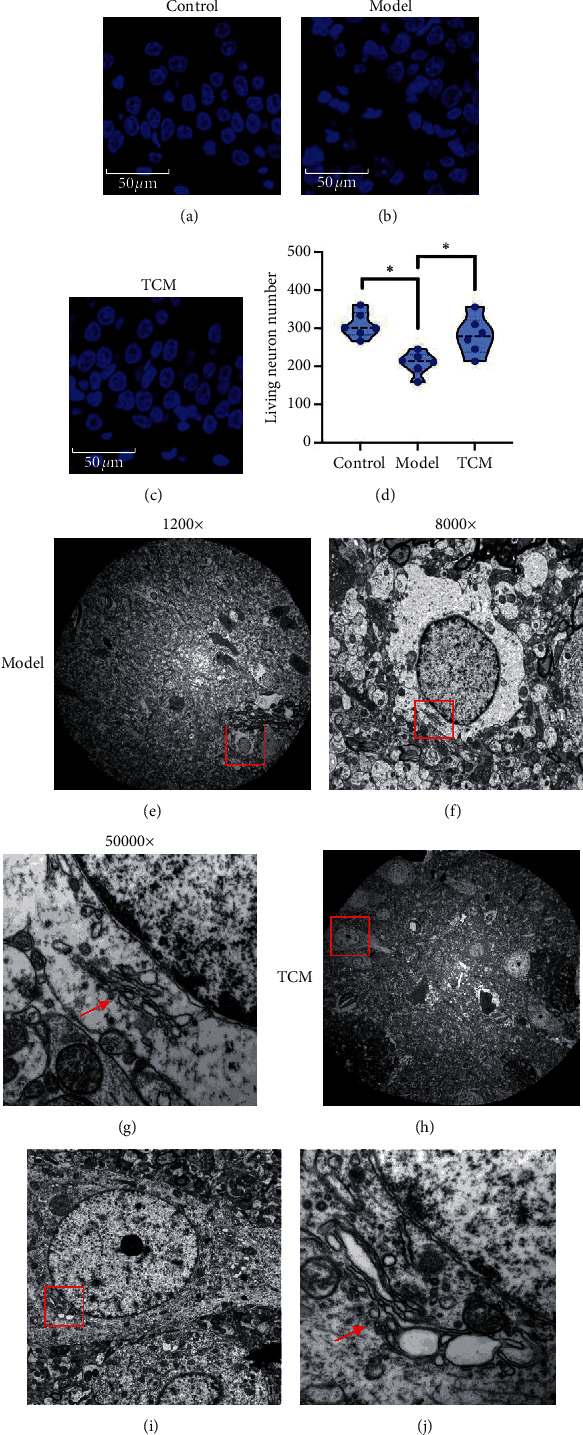
Numbers of neurons and morphological observations of the endoplasmic reticulum. (d–h) The living neuron number in different groups by DAPI staining. (e–j) All the neurons in the field of mesh of a specimen under transmission electron microscope, where the red boxes in (f) and (i) indicate single-cell fields. The red arrows in (g) and (j) indicate the ER. The “TCM group” refers to the middle-dose group. Compared with the model group, “^∗^” indicates *P* < 0.05.

TEM and DAPI staining showed fewer apoptotic nerve cells in the middle-dose group than in the model group (Figures 4(a)–4(e) and 4(h)). Magnification and observation of the ultrastructure of ER showed that ER fragmentation, degranulation (ribosome shedding), and ER number were greatly reduced in the model group. These phenomena rarely occurred in the intervention groups. Instead, ER and mitochondria were abundant in the intervention groups, suggesting that CSG reduced the ERS of nerve cells, which restored protein synthesis to normal levels (Figures 4(f) and 4(g)–4(j)).

## 4. Discussion

Rotenone is a widely used insecticide that selectively inhibits the activity of mitochondrial respiratory chain complex I in neurons, causing degeneration and apoptosis of dopaminergic neurons. It was first used by Heikkila et al. [16] to model PD in animals. Numerous studies have shown that rotenone causes mitochondrial dysfunction [17], neuroinflammation [8], and oxidative stress [18], so it is a widely recognized drug for PD modeling.

Some research results indicate that ERS and UPR are widely involved in the pathophysiology of PD. PERK, IRE-1, and ATF6 are important molecules in the three signaling pathways of ERS. In the early stages of ERS, UPR is a protective program. However, after chronic activation, it triggers apoptosis. Under the influence of ERS, PERK separates from its molecular chaperone, GRP78, and activates it, causing downstream eIF2 phosphorylation and inactivation, which stops most protein synthesis in the cell [19, 20]. CDNF is a neurotrophic factor that can protect neurons and bind to GRP78. Its expression is correlated with that of GRP78 [21–25]. Activation of UPR requires the activation of GRP78 [26]. Study of Salganik et al. [20] has shown that GRP78 expression in the substantia nigra of 12-month-old rats is significantly lower than that of two-month-old rats. Downregulating the expression of GRP78 using small interfering RNAs (siRNAs) aggravates the toxicity of *α*-syn to dopaminergic neurons in the substantia nigra. In addition, the neurodegenerative symptoms of the animal model become more severe with the decrease in GRP78 expression. Studies have also shown that inhibiting GRP78 mRNA level reduces ERS resistance to 6-OHDA-induced neuronal damage in PD rat models, and downregulation of GRP78 hinders the activity of the proapoptotic factor caspase-3, thereby improving rotenone-induced neuronal damage in the PD rat model [27, 28]. Other researchers have shown increased GRP78 expression in autopsied brain tissue of patients with PD dementia and Lewy body dementia [29]. The above findings indicate that ERS is involved in the process of PD. Regulation of the expression of GRP78, a marker protein of ERS, is one of the approaches to reduced ERS damage to cells.

In this study, GRP78 expression was downregulated in the rotenone-induced PD model. After 14 days of CSG interventions, the expression of GRP78 in the substantia nigra of the midbrains of rats in middle- and high-dose intervention groups was significantly upregulated. Expression of the key functional enzyme, TH, and CDNF in dopaminergic neurons was increased, while the JNK and phosphorylated c-JUN expression in the substantia nigra of the midbrains of rats and the apoptosis were significantly reduced. TEM study on the ultrastructure of ER showed that CSG improved ER fragmentation and degranulation in neurons of the PD rat model. In addition, the numbers of ER and mitochondria in the area of substantia nigra of the PD rat model were increased after CSG intervention, suggesting that the intracellular life activity of neurons affected by neurotoxin was recovered. Given the close relationship between GRP78-downregulated ERS and the CDNF and JNK pathways, this study demonstrated that CSG may reduce ERS by increasing GRP78 expression and reducing ERS level in the substantia nigra, thereby triggering the protective effects on the PD rat model.

## Figures and Tables

**Table 1:** Drug-dose combination orthogonal design and efficiency calculation table L8 (27).

Experiment ID	*Cistanche deserticola*	Refined *Rhizoma polygonati*	*Salvia miltiorrhiza*	*Radix Paeoniae Rubra*	Tree peony bark	Mean cell viability (*n* = 3)
A	1	1	1	1	1	143.00 ± 11.79%
B	1	1	1	2	2	133.00 ± 6.25%
C	1	2	2	1	1	150.67 ± 5.13%
D	1	2	2	2	2	143.33 ± 6.03%
E	2	1	2	1	2	128.00 ± 7.55%
F	2	1	2	2	1	134.33 ± 13.65%
G	2	2	1	1	2	117.67 ± 8.74%
H	2	2	1	2	1	132.67 ± 2.89%

Note: different levels of individual herbs: *C. deserticola* 1 = 6 g and 2 = 10 g; refined *R. polygonati* 1 = 12 g and 2 = 15 g; *S. miltiorrhiza* 1 = 12 g and 2 = 15 g; *R. Paeoniae Rubra* 1 = 12 g and 2 = 15 g; and tree peony bark 1 = 10 g and 2 = 15 g.

**Table 2:** Comparison of the therapeutic effects of CSG of different dose combinations on MPP^+^-induced *in vitro* model.

Chinese herbal medicine	Dose level	Number of experiments	Mean ± standard deviation	Confidence interval		*F*	*P*
*Cistanche deserticola*	1	3	142.50 ± 9.32	137.55	147.45	18.51	<0.01
	2	3	128.17 ± 10.28	123.22	133.12		

Refined *Rhizoma polygonati*	1	3	134.58 ± 10.41	129.63	139.53	0.20	0.69
	2	3	136.08 ± 13.96	131.13	141.03		

*Salvia miltiorrhiza*	1	3	131.58 ± 11.70	126.63	136.53	5.07	0.04
	2	3	139.08 ± 11.70	134.13	144.03		

*Radix Paeoniae Rubra*	1	3	134.83 ± 15.30	129.88	139.78	0.09	0.77
	2	3	135.83 ± 8.37	130.88	140.78		

Tree peony bark	1	3	140.17 ± 11.06	135.22	145.12	8.42	0.01
	2	3	130.50 ± 11.45	125.55	145.45		

Model *R*^2^ = 0.642.

**Table 3:** Repeated measures analysis of variance of suspension experiment (mean ± standard deviation).

Group	*N*	Before treatment	5 d	10 d	14 d	Comparison of different timepoints in the same group	
						*F*	*P*
Control	9	2.30 ± 0.51	2.22 ± 0.46	1.92 ± 0.32	1.70 ± 0.13	15.55	<0.01
Solvent	9	2.37 ± 0.48	2.23 ± 0.49	1.91 ± 0.36	1.57 ± 0.21	24.34	<0.01
Model	9	0.37 ± 0.11	0.39 ± 0.10	0.43 ± 0.17	0.47 ± 0.12	2.62	0.116
Low dose	9	0.34 ± 0.08	0.41 ± 0.85	0.61 ± 0.16	0.69 ± 0.15	55.87	<0.01
Middle dose	9	0.44 ± 0.12	0.51 ± 0.15	0.84 ± 0.05	0.87 ± 0.11	44.04	<0.01
High dose	9	0.38 ± 0.08	0.48 ± 0.09	0.81 ± 0.05	0.88 ± 0.07	95.63	<0.01
Comparison of groups at the same timepoint	F	101.41	89.78	80.39	113.39	-	-
	*P*	<0.01	<0.01	<0.01	<0.01	-	-

**Table 4:** Intra- and intergroup variance of suspension experiments.

Source of variation		SS	df	MS	*F*	*P*
Intragroup variation	Time	0.11	1.41	0.08	1.90	0.17
	Time*∗*group	9.04	7.03	1.29	32.01	<0.01
	Error	2.71	67.45	0.04		
Intergroup variation	Group	105.21	5.00	21.04	112.61	<0.01
	Error	8.97	48.00	0.19		

SS: sum of square; df: degree of freedom; MS: mean square.

## Data Availability

All data in this manuscript are available from the corresponding author for reasonable reasons and research needs.
